# Determination of Care Burden of Caregivers of Patients with Multiple Sclerosis in Turkey

**DOI:** 10.1155/2018/7205046

**Published:** 2018-03-20

**Authors:** Serpil Özmen, Afife Yurttaş

**Affiliations:** ^1^Narman Technical College, Atatürk University, Erzurum, Turkey; ^2^Faculty of Nursing, Atatürk University, Erzurum, Turkey

## Abstract

The aim of this study was to determine the care burden of caregivers of patients with multiple sclerosis in Turkey. This descriptive study was conducted with 92 caregivers. To collect data, information form and Zarit Caregiver Burden Interview (ZCBI) were used. Most of the caregivers (65.2%) were females and 71.7% of them were married. The average age of caregivers was 38 and above. The mean ZCBI score of caregivers was 25.44 ± 9.50. The ZCBI score was significantly higher in caregivers providing care for more than six years (28.09 ± 10.16). Additionally, the ZCBI score was significantly higher in caregivers providing care 3-4 hours per day (32.23 ± 8.37) and providing physical care (29.28 ± 5.18).

## 1. Introduction

Multiple sclerosis (MS) is a chronic demyelinating disease of the central nervous system, which affects the brain, the spinal cord, and the optic nerves. MS does not directly affect the peripheral nerves [1]. Diagnostic criteria for MS have evolved over the past 50 years [2–5]. In the early 1900s, a few of MS have been reported, which is an admission to neurological clinics [6]. In 1996, the US National Multiple Sclerosis Society Advisory Committee on Clinical Trials in MS defined the clinical subtypes of MS. The committee standardized four subtypes of MS: relapsing-remitting, primary-progressive, secondary-progressive, and progressive-relapsing [7]. This disease affects 2.5 million people around the world and approximately 35,000 people in Turkey [8].

At the time of diagnosis, many patients with MS are in stable relationships, which are inevitably affected by the advancing disease. The caregivers have to cope with not only the presence of the disease but also the added fact of an unpredictable prognosis, including the possibility that their caregivers may become severely physically and cognitively impaired [9–11]. Also, there are a lot of reasons increasing burden of caregivers of patients with MS in our country. In Turkey, patients and caregivers cannot benefit adequately from the primary healthcare system due to a lack of trained personnel with expertise in MS. It gives care for patients with MS in hospitals and rehabilitation centers. However, caregivers cannot benefit from these centers due to various reasons. These reasons include the limited financial of patients and their families, difficulty of reaching hospitals in these provinces and a high patient population. Both patients with MS and their caregivers may experience financial difficulties due to having a chronic disease and living in the same home [10–12]. Therefore, emotional, physical, and financial support should be given to caregivers [13–15].

Besides these, due to the cultural structure of Turkish society, usually the family members are responsible for providing care to patients. On the other hand, although care was provided by the spouses of male patients with MS, unfortunately husbands of female patients do not take responsibility for the care of their wives. Moreover, male caregivers sometimes get divorced from their wives, fearing that they cannot have children and fulfill family responsibilities. This especially increases the burden of care of the female patients with MS and their families [16].

As professionals, nurses have an important role in the implementation of care in healthcare services. It is necessary for nurses to establish healthy or nurse-patient relationship on well-established bases so that nurses can give meaningful care to healthy individuals or their patients and their families in frequently changing health practices [17].

It is thought that the level of burnout will decrease and living standards will improve as a result of the social support provided to relatives of patients with MS. Nurses have very important responsibilities in order to develop a network of social support between relatives of patients. Informing caregivers of patients with MS about their care, training them to provide care, and teaching coping strategies may reduce the burnout of caregivers [16].

Many of the previous studies have focused on the care and quality life of patients with MS, but there are a limited number of studies investigating the care burden of caregivers of patients with MS in Turkey. Therefore, this study was conducted to determine the care burden of caregivers of patients with MS in Turkey. We think that this research will contribute to the literature.

## 2. Materials and Methods

### 2.1. Participants and Recruitment Procedure

**Figure 1:**
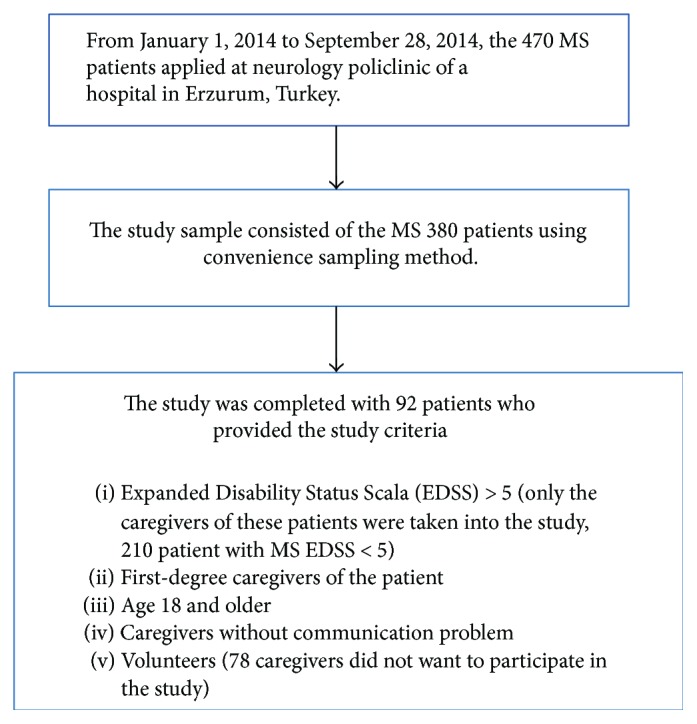
The flowchart of the study.

This study was conducted at neurology policlinic of a hospital in Erzurum, Turkey, between January 1, 2014, and September 28, 2014. Between these dates, the 470 MS patients applied to the neurology policlinic. The study sample consisted of 380 patients using sampling method [18]. The caregivers were selected through convenience sampling. Inclusion criteria were as follows: Expanded Disability Status Scale (EDSS) > 5 (only the caregivers of these patients were taken into the study), first-degree caregivers of the patient, age of 18 and older, caregivers without communication problem, and volunteers. Eventually, the study was completed with 92 patients with MS.  Figure 1 shows the flowchart of the study.

### 2.2. Ethical Consideration

Caregivers were informed about the purpose of this study upon inclusion in the database and consent was obtained after oral and written information.

The local ethical committee of the University of Atatürk, Erzurum, Turkey, approved the study (IRB; AU 2014.12.2/b). The study was conducted according to the Declaration of Helsinki.

### 2.3. Measurements

#### 2.3.1. The Information Form

The information form was prepared by the researchers using the literature [19–29]. In the information, there were questions including age, gender, marital status, educational status, and problems experienced during care.

#### 2.3.2. Zarit Caregiver Burden Interview (ZCBI)

The ZCBI was developed by Zarit and colleagues in 1980. It consists of 22 items. In its 22-item version, the highest score is 88 and lowest score is 0. Higher scores indicate greater burden. The ZCBI measures subjective burden in terms of the degree to which the caregiver experiences physical, psychological, emotional, social, and financial problems as a result of their caregiving role. The Cronbach's alpha of ZCBI ranges from 0.85 to 0.94 [30].

The Turkish validity and reliability of the scale was performed by Ozer et al. in 2012 and the Cronbach's alpha value is in the range of 0.82 [31]. Our research Cronbach's alpha value is 0.85.

Many studies have been carried evaluating the care burden of caregivers of patients in the world and Turkey. The majority of these studies have used ZCBI [19–29].

### 2.4. Data Collection/Procedure

An information form and ZCBI were administered to the caregivers in a patient education class of neurology service. The instructions were made using face-to-face interviews by researchers. The caregivers were asked to mark the most suitable statement for each item of the scale and the information form. It took approximately 30–35 minutes responding the information form and ZCBI.

### 2.5. Data Analysis

Data analysis was performed by using the Statistical Package for the Social Sciences (SPSS) 16 packet program. We used Kruskal-Wallis test and Mann–Whitney *U* test to compare continuous variables in independent groups. *P* < 0.05 for the results was considered statistically significant.

## 3. Results

A large part of caregivers enrolled in the present study were women, married, and unemployed; most of them had children and some of them a relatively high number of children. Half of the caregivers had a social insurance. In addition, one in four of them was the spouse of the patient. A large of caregivers lived with the patient (Table 1).

The vast majority of caregivers had information about MS. In addition, more than half of them had information about MS treatment and care. Approximately one-third of them gave care more than six years to patient with MS patient and one-third of the caregivers gave this care at the patient's home. A large part of caregivers did not find enough this care. Most of the caregivers gave physical, social, and economic care to patient with MS (Table 2).

The mean scores of caregivers who had social insurance from the pension fund were higher than the other groups (27.61 ± 10.71). The mean scores of the caregivers who were spouses of the patients were higher than the other groups (27.16 ± 10.62). The mean scores of the caregivers who did not live with the patient (26.56 ± 8.85) were higher (Table 3).

The mean scores of the caregivers who received MS-related information (25.83 ± 8.82), who received this information from healthcare worker (26.13 ± 9.00), and who found the received information sufficient (25.87 ± 8.18) were higher and no statistically significant difference was found between the mean scores. The mean scores of the caregivers, who were care providing their patients for more than 6 years, (28.09 ± 10.16) were higher. The mean scores of the caregivers who provided care at the patient's house (26.00 ± 11.33) were higher (Table 4).

The mean scores of caregivers who provided care for 3-4 hours per day (32.23 ± 8.37) and provided physical care (29.28 ± 5.18) were higher. The mean scores of the caregivers, who did not consider the time spent for care sufficient (36.25 ± 12.14) and who expressed that more than one person provided support for the care (26.95 ± 10.86), were higher than the other groups (Table 4).

## 4. Discussion

In the study, it was found that the majority of caregivers were females. This result can be interpreted to say that caregiving is traditionally known a responsibility of the woman and also women consider caregiving the continuation of their older responsibilities, whereas men are strangers to the caregiving responsibility. Studies have revealed that the majority of caregivers were women and patient's spouses [32, 33].

The majority of caregivers lived in the same house with the patient. In addition, majority of caregivers had children and majority of them had 4-5 family members living with them. This may be associated with the fact that the caregivers were mostly patients' parents or spouses. In a similar study, it was found that the majority of caregivers and patients lived in the same house [34].

When ZCBI scores were compared to each other according to the caregivers' status of living with patients in the same house, the mean scores of the caregivers living in separate environments and who had 4-5 family members living with them were higher. This may make us think that the caregivers who lived in the same house environment with their patients may be more useful in terms of the continuity of the care. This result of our study shows similarity with the results of other studies in the literature [24, 35].

The minority of the caregivers included in the study provided care also for other individuals in need of care and these persons were the caregivers' parents or spouses. In a similar study reported that the caregivers also provided other people with care and the individuals they provided care for were their parents, spouses, and children [36].

It was determined that 45.0% of caregivers provided the patients with care for 1–5 years and 1-2 hours per day and they found the time spent for the care sufficient. Acccording to the study conducted by National MS Society in 2012, it was found that while 35.0% of caregivers provided their patients with care for 1–10 hours, 3% provided the care for 81 hours and above [37]. In another study, 50% of caregivers provided their patients with care for 1–5 hours [38].

It was determined that majority of the caregivers supported their patients from psychological, social, economic, and physical aspects, whereas minority of them supported only the physical needs of their patients. Carod-Artal et al. found that those who provided primary care within the care process gave support to their patients mostly in terms of coping with the disease and in psychological aspects [28]. It was also reported in another study that patients not only felt physically incompetent but also were affected in psychosocial aspect and supporting the patients in psychosocial aspect may be important in order to increase their life qualities [29].

The mean scores received by the employed patient relatives, included in the study, from the ZCBI were found to be higher compared to patient relatives who were unemployed. This situation may be associated with the fact that the caregiver had difficulties in the caregiving procedure due to the additional roles added to the roles of the caregiver. Zarit et al. emphasized that employment caused stress on caregivers, and accordingly, risk of depression may increase among caregivers; in addition, caregivers may feel exhausted and tired, and serious increase in health problems such as insomnia, diabetes, and arthritis and decrease in social bonds may be present [30].

The mean scores received by the participating caregivers aged 38 and over from the ZCBI were found to be higher compared to the other age groups. In contrast to this information, the studies have reported that age affects the care burden and younger caregivers experience heavier burdens [31].

When the mean score received from ZCBI was examined in terms of gender and marital status, it was observed that patient relatives who were males and married had higher mean scores. In the study entitled “Caregiver burden among informal caregivers assisting people with multiple sclerosis” conducted by Buchanan et al., it was found that the burden of male caregivers was higher than women [32]. Contrary to this, another study reported that the risk of experiencing emotional burden was higher among women compared to men and their life qualities were lower than men [33].

Among the caregivers included in the study, the care burden mean scores of spouses were found to be higher than other relatives. Previous studies reported that care was mostly provided by spouses and this process was considered as limiting the social life for both men and women. Also, disorders such as depression and anxiety were observed to be increasing in spouses during the process of caregiving [34, 35]. Figved et al. found that the spouses providing the care to MS patients experienced more distress compared to other groups and their life qualities were lower [36].

It was found that the mean scores of the caregivers, providing their patients with care for six years and longer during the care process, were higher than other groups, which caused a significant difference. Özyeşil et al. [37] indicated that there was a positive correlation between continuous and long care process and the developmental burden, physical burden, social burden, and emotional burden of caregivers.

Among caregivers included in this study, the mean scores of those who had any disease were higher than healthy caregivers, which caused a significant difference. In the study of Taşdelen and Ateş [38], it was found that half of the caregivers had a chronic disease. The study by Özmen and Yurttaş showed that the majority of caregivers had a disease [39]. Since caregivers devote themselves to the care procedure, it can be said that they may neglect their own diseases and treatments. Consequently, providing someone else with care in addition to their own health problems may create burden on caregivers.

The results of the study are limited to caregivers of patients with MS at the university hospital where the study was conducted. Furthermore, other limitations of the study include a small sample and a single center.

## 5. Conclusions

In conclusion, our study showed that the ZCBI score increased as the age of caregivers increased. The caregivers who care for longer than six years had a higher ZCBI mean scores. In line with these results, caregivers' burden and affecting factors should be identified in future research with a large sample. The nursing care should include not only patients but also their caregivers. Additionally, information and education about MS should also be given to caregivers.

## Figures and Tables

**Table 1:** Demographic characteristics of caregivers.

Demographic characteristics	*n*	%
*Age*		
18–27	27	29.4
28–37	22	23.9
38 or above	43	46.7
*Gender*		
Female	60	65.2
Male	32	34.8
*Education level*		
Literate	12	13.0
Primary school	45	48.9
High school	11	12.0
University	24	26.1
*Occupation*		
Working	32	34.8
Non-working	60	65.2
*Working status*		
Worker	19	59.3
Officer	13	40.7
*Marital status*		
Married	66	71.7
Single	26	28.3
*Having children*		
Yes	61	66.3
No	31	33.7
*Number of children* (*n* = 61)		
1-2	22	36.1
3-4	23	37.7
5 and above	16	26.2
*Social insurance*		
Green card	18	19.6
Social insurance	50	54.3
Pension fund	18	19.6
Self-employed	6	6.5
*The degree of proximity to patient*		
Parents	29	31.5
Spouse	25	27.2
Son/daughter	22	23.9
Sibling	16	17.4
*Living situations with patients*		
Yes	69	75.0
No	23	25.0
*The number of family members living together*		
2-3	30	32.6
4-5	37	40.2
6 and above	25	27.2
*Having any disease*		
Yes	17	18.5
No	75	81.5

**Table 2:** Level of information about MS of the caregiver sample.

Features	*n*	%
*Having information about MS*		
Yes	60	65.2
No	32	34.8
*Having information about MS treatment*		
Yes	56	60.9
No	36	39.1
*Need information about MS care*		
Yes	51	55.4
No	41	44.6
*Getting information about MS*		
Yes	55	59.8
No	37	40.2
*Who did give your information about MS?* (*n* = 55)		
Healthcare worker	51	92.7
Internet	4	7.3
*Is your information enough?* (*n* = 55)		
Yes	40	72.7
No	15	27.3
*Duration of care given for MS*		
Less than 1 year	17	18.5
1–5 years	42	45.7
More than 6 years	33	35.8
*Where did you give MS care?*		
At hospital	3	3.3
At home of patients with MS	36	39.1
At home of caregivers	32	34.8
Both at hospital and at home	21	22.8
*Duration of care given to the patient*		
1-2 hours	38	41.3
2-3 hours	25	27.2
3-4 hours	17	18.5
4-5 hours	8	8.7
All day	4	4.3
*Do you find enough time spending for care?*		
Yes	88	95.7
No	4	4.3
*Which areas do you care?*		
Psychological	8	8.7
Physical	7	7.6
Psychological, social, and economic	77	83.7

**Table 3:** ZCBI mean scores ± SD as a function of demographic characteristics of caregivers and summary of statistical comparisons.

Features	*X* ± SD	Test and *P* value
*Age*		
18–27	22.70 ± 8.26	
28–37	24.36 ± 9.78	KW = 4.97
38 or above	27.72 ± 9.73	*P* > 0.05^∗^
*Gender*		
Female	24.88 ± 9.50	*t* = 0.77
Male	26.50 ± 9.56	*P* > 0.05
*Education level*		
Literate	28.58 ± 6.69	
Primary school	26.73 ± 10.37	KW = 6.01
High school	24.45 ± 9.22	*P* > 0.05
University	21.91 ± 8.43	
*Occupation*		
Working	23.68 ± 7.21	*t* = 1.30
Non-working	26.38 ± 10.45	*P* > 0.05
*Working status*		
Worker	23.89 ± 7.57	*t* = 0.19
Officer	23.38 ± 6.93	*P* > 0.05
*Marital status*		
Married	26.39 ± 9.30	MW-U = 672.00
Single	23.03 ± 9.75	*P* > 0.05
*Having children*		
Yes	27.29 ± 9.41	*t* = 2.70
No	21.80 ± 8.71	*P* < 0.05
*Number of children* (*n* = 61)		
1-2	28.22 ± 10.95	
3-4	26.13 ± 9.96	KW = 0.78
5 and above	27.68 ± 6.11	*P* > 0.05
*Social insurance*		
Green card	27.55 ± 11.45	
Social insurance	23.92 ± 7.77	KW = 2.05
Pension fund	27.61 ± 10.71	*P* > 0.05
Self-employed	25.33 ± 12.59	
*The degree of proximity to patient*		
Parents	24.82 ± 8.03	
Spouse	27.16 ± 10.62	KW = 0.60
Son/daughter	26.68 ± 9.89	*P* > 0.05
Sibling	22.18 ± 9.50	
*Living situations with patients*		
Yes	25.07 ± 9.74	MW-U = 724.00
No	26.56 ± 8.85	*P* > 0.05
*The number of family members living together*		
2-3	25.40 ± 8.77	KW = 0.19
4-5	26.37 ± 11.02	*P* > 0.05
6 and above	24.12 ± 7.99	
*Ownership of any disease*		
Yes	29.23 ± 8.28	MW-U = 419.50
No	24.58 ± 9.60	**P** < 0.05

^∗^NS = not significant.

**Table 4:** ZCBI mean scores ± SD as a function of level of information about MS and summary of statistical comparisons.

Features	*X* ± SD	Test and *P* value
*Having information about MS*		
Yes	25.31 ± 9.15	*t* = 1.17
No	25.68 ± 10.26	*P* > 0.05^∗^
*Having information about MS treatment*		
Yes	25.21 ± 9.04	*t* = 0.29
No	25.80 ± 10.30	*P* > 0.05
*Need information about MS care*		
Yes	26.03 ± 10.6	*t* = 0.66
No	24.70 ± 7.85	*P* > 0.05
*Getting information about MS*		
Yes	25.83 ± 8.82	*t* = 0.47
No	24.86 ± 10.52	*P* > 0.05
*Who did give your information about MS?* (*n* = 55)		
Healthcare worker	26.13 ± 9.00	MW-U = 65.00
Internet	22.00 ± 5.35	*P* > 0.05
*Is your information enough?* (*n* = 55)		
Yes	25.87 ± 8.18	MW-U = 283.50
No	25.73 ± 10.67	*P* > 0.05
*Duration of care given for MS*		
Less than 1 year	20.64 ± 7.76	KW = 6.79
1–5 years	25.30 ± 8.99	**P** < 0.05
More than 6 years	28.09 ± 10.16	
*Where did you give MS care?*		
At hospital	24.00 ± 7.00	KW = 0.21
At home of patients with MS	26.00 ± 11.33	*P* > 0.05
At home of caregivers	24.75 ± 8.87	
Both at hospital and at home	25.76 ± 7.56	
*Duration of care given to the patient*		
1-2 hours	22.78 ± 7.59	
2-3 hours	25.16 ± 10.77	KW = 12.95
3-4 hours	32.23 ± 8.37	**P** < 0.05
4-5 hours	22.50 ± 8.65	
All day	29.50 ± 12.39	
*Do you find enough time spending for care?*		
Yes	24.95 ± 9.15	MW-U = 77.50
No	36.25 ± 12.14	*P* > 0.05
*Which areas do you care?*		
Psychological	17.25 ± 6.34	
Physical	29.28 ± 5.18	KW = 7.72
Psychological, social, and economic	25.94 ± 9.67	**P** < 0.05

^∗^NS = not significant.

## Additional Points

This research was accepted as a master thesis (January 22, 2015).
